# Low-temperature combustion of methane over graphene templated Co_3_O_4_ defective-nanoplates

**DOI:** 10.1038/s41598-021-92165-4

**Published:** 2021-06-15

**Authors:** Dian Gong, Gaofeng Zeng

**Affiliations:** 1 CAS Key Laboratory of Low-carbon Conversion Science and Engineering, Shanghai Advanced Research Institute, Chinese Academy of Sciences, 100 Haike Road, Shanghai, 201210 China; 2 School of Chemical Engineering, University of Chinese Academy of Sciences, 19A Yuquan Road, Beijing, 100049 China

**Keywords:** Catalysis, Environmental chemistry, Materials chemistry, Materials for energy and catalysis, Nanoscale materials, Graphene

## Abstract

Transition metal oxides are the potential catalysts to replace noble-metal based catalyst for the catalytic combustion of methane due to the tolerable reactivity and low cost. However, these catalysts are challenged by the low temperature reactivity. Herein, the surface defective Co_3_O_4_ nanoplates are realized through a facile co-precipitation and thermal reduction method with the association of GO. The resultant catalysts (CoGO50) demonstrate a superior low-temperature reactivity for the methane oxidation to CO_2_ and H_2_O in comparison with the common Co_3_O_4_ catalyst. The reliable stability of CoGO50 catalyst was proved by 80 h testing with intermittent feeding of water vapor. The experimental analysis demonstrates that the presence of a small amount of GO significantly affects the catalysts in surface valence state, active oxygen species and surface oxygen vacancies through reacting with the cobalt oxide as a reductant. Moreover, GO plays as 2D confine template to form smaller and thinner nanoplates. This work provides a facile method to control the surface properties of catalyst not only for Co_3_O_4_ based catalysts but also for wider solid catalysts.

## Introduction

The emission of low content methane (CH_4_) from mining, livestock and heavy engines poses a serious threat to our environments because that CH_4_ is a strong greenhouse gas with 22 times higher effect than that of equal amount carbon dioxide^1^. Conventionally, the low content methane is treated by flame combustion at > 1000 °C, which results in the generation of poisonous gases like NO_x_ and CO^2,3^. Therefore, catalytic combustion of methane, which can convert methane into innoxious CO_2_ and H_2_O at relatively low temperature (< 600 °C), has attracted many attentions^4^. Supported noble metal catalysts, such as Pt, Au, Rh and Pd, are widely studied for the methane combustion, which can efficiently reduce the reaction temperature even to ~ 300 °C^5–8^. However, these catalysts are challenged by high cost and easy poisoning^6^. Therefore, there are many interests on developing non-precious metal based catalyst. As alternatives, the low cost transition metal oxides (TMOs), including cobalt oxides^9^, manganese oxides^10^ and nickel oxides^11^, exhibit potential activity for methane catalytic combustion. In comparison with noble metal catalysts, however, TMOs catalysts are still limited the relatively high conversion temperature^12,13^. Therefore, it is desirable to develop efficient method to enhance the reactivity of TMOs based catalyst for methane activation at relatively low temperature.

Graphene and it derivatives have atomic 2D structure with unique electronic and chemical properties, which have become versatile materials in many applications including catalysis^14,15^. With the help of dislocations, vacancies, edges, impurities and functional groups, importantly, graphene based catalysts exhibit improved reactivity for the catalytic oxidation reactions at low temperature^16^. In our previous work, we found that the ultrathin graphene shell on V_2_O_5_/TiO_2_ can enhance the surface acidity of catalyst, leading to improved reactivity for the methanol oxidation at low temperature^17^. Moreover, graphene matrix fixed single iron atoms offered impressive activity in the oxidation of benzene and methane even at room temperature^18,19^. 3D structured Co_3_O_4_/graphene oxide (GO) catalyst exhibited low-temperature CO oxidation activity^20^. On the other hand, Co_3_O_4_ nanoparticles have been considered as one of the most efficient TMOs catalysts for methane combustion. It has also been proved that 2D nanoplate Co_3_O_4_ offer higher activity for methane catalytic combustion than the 0D nanoparticle and 1D nanofiber due to the exposed high-index planes of Co_3_O_4_ nanoplate^21,22^. Therefore, it is highly potential to further improve the reactivity of Co_3_O_4_ nanoplate catalyst by using graphene associated preparation method.

In this work, we developed graphene oxide involved Co_3_O_4_ catalyst (CoGO) through a facile co-precipitation method. The low temperature activity of the resultant catalyst was highly improved, which exhibits 100 °C decrease for the complete conversion of methane in compared with the common Co_3_O_4_. Effects of GO addition on the structure and surface chemistry of the catalyst as well as the reaction stability were thoroughly investigated.

## Results

### Effects of GO on the structure and texture of catalysts

**Figure 1:**
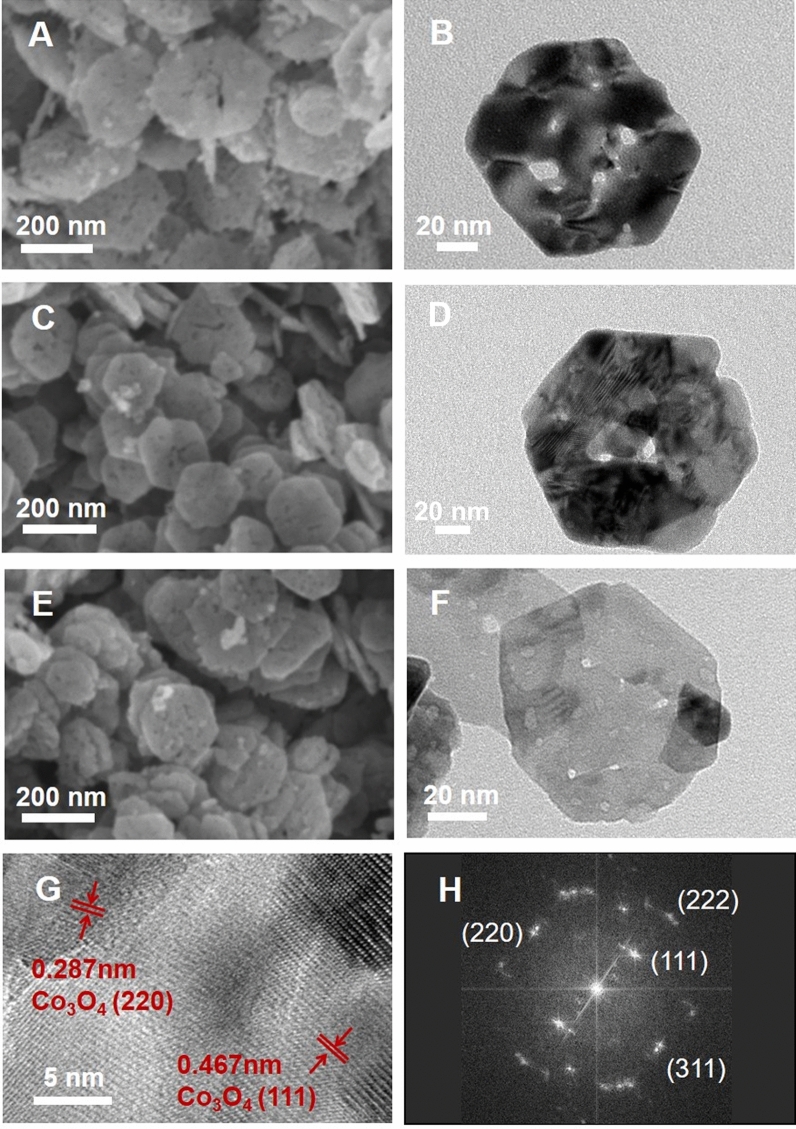
FE-SEM images (**A**,**C**,**E**) and TEM images of (**B**,**D**,**F**) of Co_3_O_4_ (**A**,**B**), CoGO100 (**C**,**D**) and CoGO50 (**E**,**F**). (**G**) HRTEM image and (**H**) the corresponding SAED pattern of CoGO50.

The scanning electron microscope (SEM) images show that all samples of Co_3_O_4_, CoGO50 and CoGO100 have the hexagonal plate-like morphology (Fig. 1A,C,E). Noted that the precursor of Co_3_O_4_, i.e., CoOOH, also possesses the hexagonal nanoplate morphology, suggesting that the shape of Co_3_O_4_ was well maintained upon the calcination (Figure S1). The average sizes of CoGO20, CoGO50 and CoGO100 nanoplates (~ 150–250 nm) are slightly smaller than that of Co_3_O_4_ (~ 300 nm) (Figs. 1 and S2). This suggests that GO addition not only protects the plate shape of Co_3_O_4_ but also confines the growth of Co_3_O_4_ crystals. The transmission electron microscope (TEM) images of single nanoplate of Co_3_O_4_, CoGO50 and CoGO100 display that the thickness of CoGO50 is thinner than others, which may contribute to the confinement effect of GO flakes in the preparation (Fig. 1B,D,F). In addition, no graphene layers were found from both CoGO50 and CoGO100, suggesting that GO has been consumed in the calcination of catalysts.

The high-resolution TEM (HRTEM) image of CoGO50 shows an interplanar spacing of 0.287 nm, corresponding to the cubic Co_3_O_4_ (220), and an interplanar spacing of 0.467 nm, assigned to the cubic Co_3_O_4_ (111) plane (Fig. 1G). The microstructure of CoGO50 is well consistent with that of Co_3_O_4_ (Figure S3). Consistently, no lattice fringe of graphic carbon was observed from the HRTEM image of CoGO50 (Fig. 1G). The corresponding selected-area electron diffraction (SAED) pattern reveals the typical diffraction spots of hexagonal Co_3_O_4_, suggesting a high crystallinity of CoGO50 (Fig. 1H).

**Figure 2:**
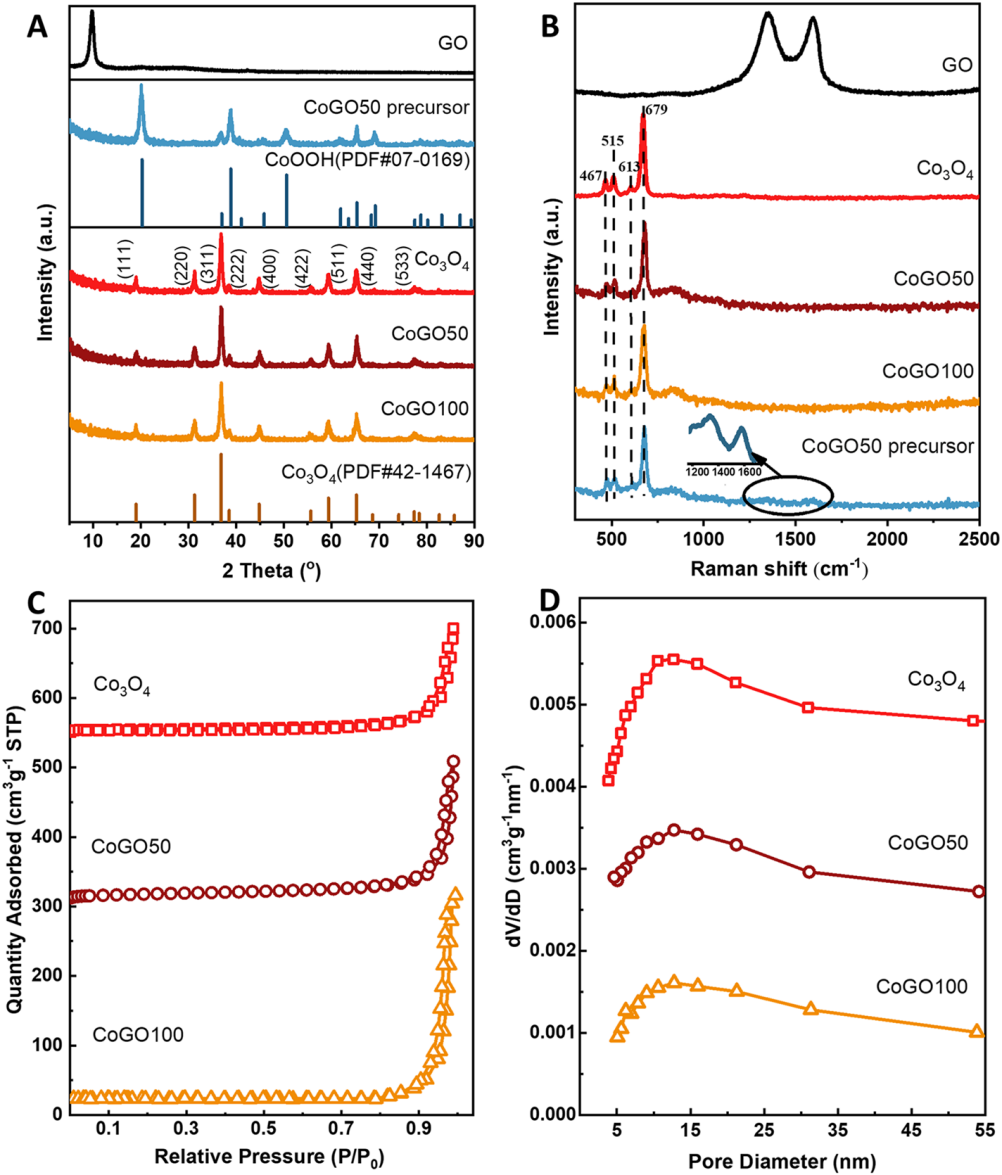
(**A**) XRD patterns and (**B**) Raman spectra of GO, Co_3_O_4_, CoGO50 and CoGO100; (**C**) Nitrogen adsorption–desorption isotherms and (**D**) the pore distribution of Co_3_O_4_, CoGO50 and CoGO100.

The crystalline structure of the catalysts was measured by X-ray diffraction (XRD), as shown in Fig. 2A. Before calcination, CoGO50 precursor exhibits the crystal structure of CoOOH (JCPDS PDF#07-0169). In addition, no signals of GO were detected from CoGO50 precursor, suggesting that GO is highly dispersed on the CoOOH surface with ultrathin thickness. The patterns of Co_3_O_4_, CoGO50 and CoGO100 show the same diffraction peaks at 2θ = 19.0°, 31.3°, 36.9°, 38.5°, 44.8°, 55.7°, 59.4°, 65.2°, and 77.3°, which are assigned to (111), (220), (311), (222), (400), (422), (511), (440), and (533) lattice planes of face cantered cubic Co_3_O_4_ (JCPDS PDF# 42-1467), respectively^23^. Similarly, no peaks of GO (~ 12°) or graphitic carbon (~ 25°) were observed from CoGO50 and CoGO100, indicating that GO was decomposed in the calcination or the amount of residual GO/GO-derivatives is beyond the detection limit^24^.

The structure of samples was further investigated by Raman spectroscopy (Fig. 2B). The spectrum of GO contains a G band at 1595 cm^−1^, arising from the first-order scattering of sp^2^ carbon atoms in a 2D hexagonal lattice, and a D band at 1343 cm^−1^, ascribed to the vibrations of carbon atoms in plane terminations of disordered graphite^25,26^. For the CoGO50 precursor, the characteristic D and G bands of GO with weak intensities were also observed (Fig. 2B inset), indicating that the surface covered GO is ultrathin. This is consistent with the XRD results that GO is highly dispersed on the CoOOH surface. The spectra of CoGO50 and CoGO100 post peaks at 467 cm^−1^ (E_g_), 515 cm^−1^ (F_1g_^1^), 613 cm^−1^ (F_2g_^1^) and 679 cm^−1^ (A_1g_^1^)^27^, which are the same as that of Co_3_O_4_ spinel structure^28^. This reveals that the bulk structure of Co_3_O_4_ was well maintained for the samples of CoGO50 and CoGO100. Moreover, no signals of graphtic D and G bands were observed from both CoGO50 and CoGO100, suggesting the amount of GO or rGO is below the detection limit.

The inner structure and surface area of the samples were measured by nitrogen adsorption isotherms (Fig. 2C). All the samples showed the IV-typed sorption isotherm with H3-typed hysteresis loop in the relative pressure range of P/P_0_ = 0.5–1.0, suggesting a mesoporous structure for these samples^29,30^. The pore sizes calculated by the Barrett–Joyner–Halenda (BJH) method show a unimodal distribution centred at ~ 12.7 nm for these samples (Fig. 2D), which is consistent with the TEM observations. For CoGO50 and CoGO100, the Brunauer–Emmett–Teller (BET) specific surface areas are 39.7 and 43.2 m^2^ g^−1^, respectively, which are higher than that of Co_3_O_4_ (31.5 m^2^ g^−1^, Table S1). This indicates that the addition of GO increases the BET surface area through forming smaller and thinner nanoplates. Moreover, the samples of CoGO50 and CoGO100 also exhibit larger pore volumes (0.31 and 0.28 cm^3^ g^−1^) than that of Co_3_O_4_ (0.23 cm^3^ g^−1^), suggesting that the existence of GO layer promotes the formation of pore during the calcination.

**Figure 3:**
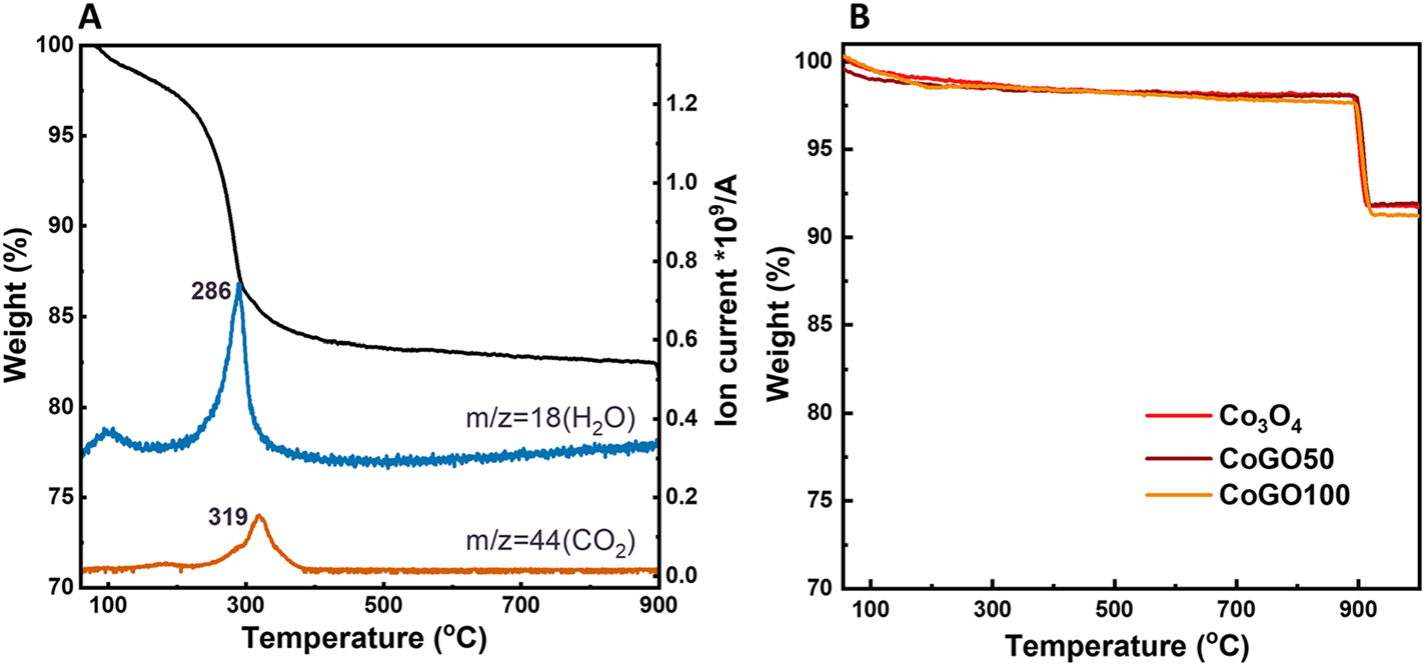
(**A**) TG-mass profiles of CoGO50 precursor in air (H_2_O signal m/z = 18 and CO_2_ signal m/z = 44) and (**B**) TG curves of Co_3_O_4_, CoGO50 and CoGO100 in air.

The thermal behaviours of the catalysts were investigated by a thermogravimetric-mass analyser (TG-MS) in the temperature range of 60–1000 °C. As shown in Fig. 3A, the CoGO50 precursor (i.e., GO covered CoOOH) exhibits weight loss mainly at ~ 110 °C and ~ 320 °C. Combined with the mass spectrometry signals, the weight loss at low temperature of 80–120 °C is ascribed to evaporation of adsorption water while the weight loss centred at 286 °C is contributed to the dehydration reaction of phase transition from CoOOH to Co_3_O_4_ as well as the thermal reduction of GO to reduced GO (rGO)^17^. Furthermore, CO_2_ signal was detected at 319 °C, which reflects the deep oxidation of GO/rGO to CO_2_ and H_2_O. It confirms that the surface covered GO was decomposed in the calcination of catalyst, in line with the observations of TEM and Raman measurements. The decomposition of rGO layer would impact the surface chemistry of Co_3_O_4_ if the surface oxygen of Co_3_O_4_ is involved in the oxidation of rGO. The thermal stability of resultant catalysts was tested by TG (Fig. 3B). Except the desorption of water at low temperature, all the samples kept stable in weight at the temperature of < 890 °C, suggesting a reliable thermal stability for these catalysts. On the other hand, TG curves show weight loss in the temperature ranges of 890–930 °C, which can be attributed to the phase transition from Co_3_O_4_ to CoO with O_2_ generation (Figure S4). The weight loss rate of this step was 6.4%, which is close to the theoretical value of O_2_ release (6.6%).

### Effects of GO on the chemistry of catalysts

**Figure 4:**
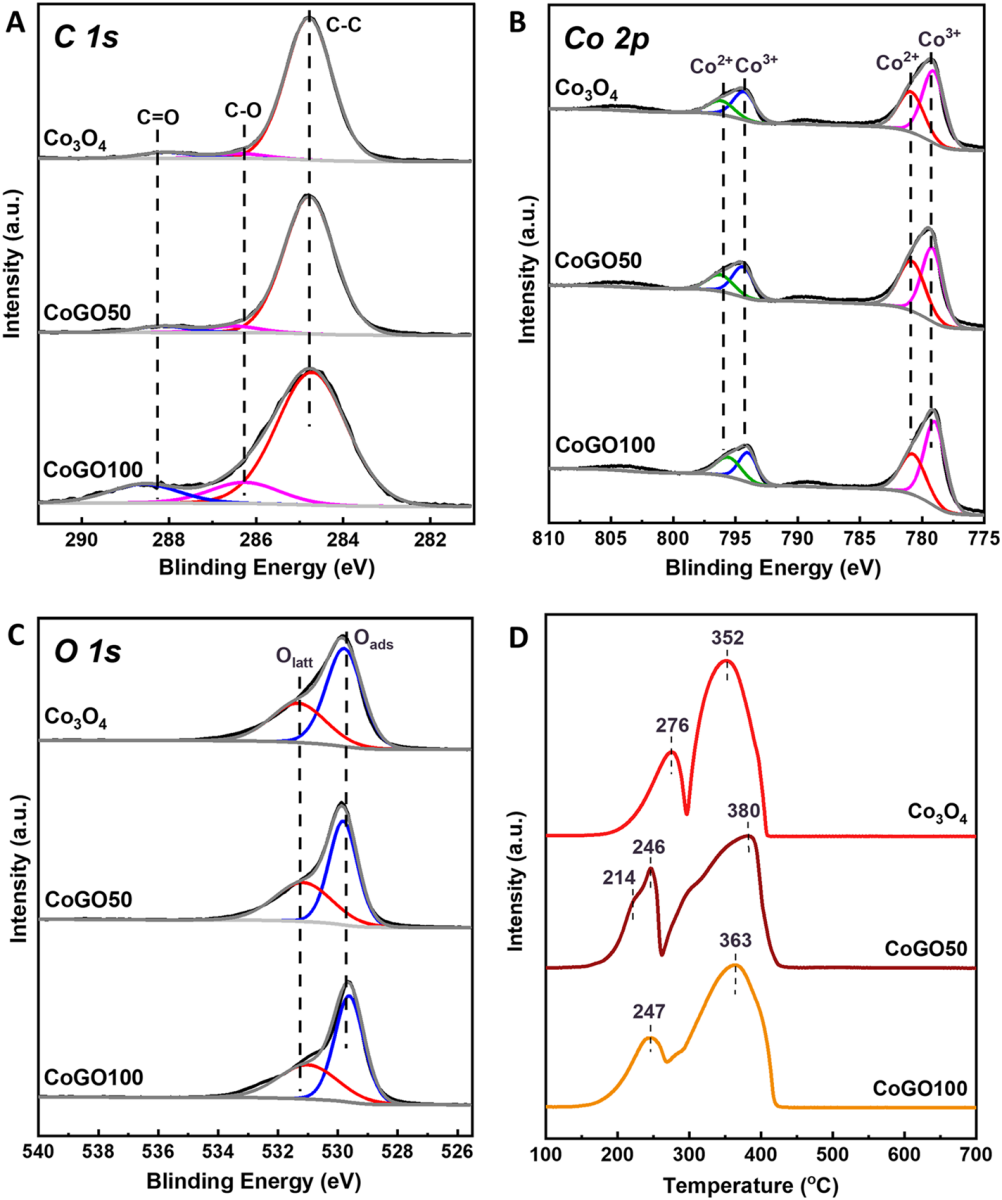
XPS C 1s (**A**), Co 2p (**B**) and O 1 s (**C**) spectra and H_2_-TPR profiles (**D**) of Co_3_O_4_, CoGO50 and CoGO100.

The near-surface electronic states of the catalysts were examined by X-ray photoelectron spectroscopy (XPS). The C1s XPS spectra of Co_3_O_4_, CoGO50 and CoGO100 display strong signal of C–C (284.8 eV) with weak signals of C–O (286.1 ± 0.2 eV) and C=O (288.2 ± 0.1 eV)^31^, as shown in Fig. 4A. The carbon signals of Co_3_O_4_ comes from the contaminations, such as conductive tape, which typically has C–C, C–O–C and O–C=O groups^31^. CoGO50 possesses similar C–O and C=O intensities to that of Co_3_O_4_, while CoGO100 has slightly higher C–O and C=O than that of Co_3_O_4_ and CoGO50. It indicates that no GO or GO derivatives were left for CoGO50 upon the calcination, in line with the observations of TEM and XRD. In comparison, CoGO100 contains trace GO residues on the surface because of higher GO concentration in the preparation.

For the twin peaks of Co 2p, the binding energies (BE) at 781.5 eV and 796.5 eV are attributed to Co^2+^ while BEs at 794.5 eV and 779.0 eV are assigned to Co^3+^ (Fig. 4B)^32^. As shown in Table 1, the surface Co^2+^/Co^3+^ ratio of CoGO50 is 0.86, which is significantly higher than that of Co_3_O_4_ (0.58) and CoGO100 (0.65). This indicates that the surface of Co_3_O_4_ is reduced by GO and its derivatives in the preparation. In the calcination of catalyst, the tightly contacted GO would be thermal-reduced firstly by losing oxygen-containing groups under the heating conditions. With higher temperature, the rGO tends to be further oxidized to CO_2_ and H_2_O, where oxygen molecules and surface oxygen of Co_3_O_4_ are involved in the oxidation reaction (Figure S5). Therefore, partial of Co^3+^ atoms on the catalyst surface would be reduced to Co^2+^ and/or oxygen vacancies would be formed on the catalyst surface^33^.

**Table 1:** Surface chemistry of Co_3_O_4_, CoGO50 and CoGO100.

Sample	Co^2+^	Co^3+^	Co^2+^/Co^3+^	O_ads_/O_latt_
Co_3_O_4_	36.61	63.39	0.58	0.61
CoGO50	46.23	53.77	0.86	0.75
CoGO100	39.21	60.79	0.65	0.65

As shown in Fig. 4C, the O1s spectra of catalysts were composed by two peaks centred at 529.7 ± 0.1 eV (lattice oxygen, O_latt_) and 531.2 ± 0.2 eV (surface adsorbed oxygen, O_ads_)^29,33,34^. The surface O_ads_/O_latt_ ratio of CoGO50 is 0.75, which is higher than that of Co_3_O_4_ and CoGO100 (Table 1). As indicated by the Co 2p results, the partial surface oxygen was consumed in the calcination, which may lead to the formation of oxygen vacancies^33^. Furthermore, the oxygen vacancies would enhance the adsorption and activation of molecular oxygen, resulting in high O_ads_/O_latt_ ratio for CoGO50. It is well known that high O_ads_/O_latt_ reflects high catalytic activity for the oxidation of hydrocarbons at low temperature^35–37^. Therefore, this suggests that CoGO50 possesses the highest reactivity for methane oxidation, which is well consistent with the following reaction test. In addition, the XPS analysis suggests that the spent CoGO50 have slightly decline in the O_ads_/O_latt_ (0.69) and Co^2+^/Co^3+^ (0.73), which are still higher than those of CoGO100 and Co_3_O_4_ (Figure S6).

The redox properties of catalysts were investigated by hydrogen temperature-programmed reduction (H_2_-TPR) (Fig. 4D). All catalysts show two reduction peaks at 200–300 °C, attributable to the reduction of Co^3+^ to Co^2+^, and at 350–400 °C, assigned to the reduction of CoO to metallic cobalt (Co^0^)^32,38^. The reduction temperature downshifted from 276 °C of Co_3_O_4_ to 214–246 °C of CoGO50. This reveals that CoGO50 contains more active oxygen species, which are much easier to be reduced. Therefore, CoGO50 would take high oxidative activity in the catalytic oxidation reaction.

### Catalytic combustion of methane over CoGO catalysts

**Figure 5:**
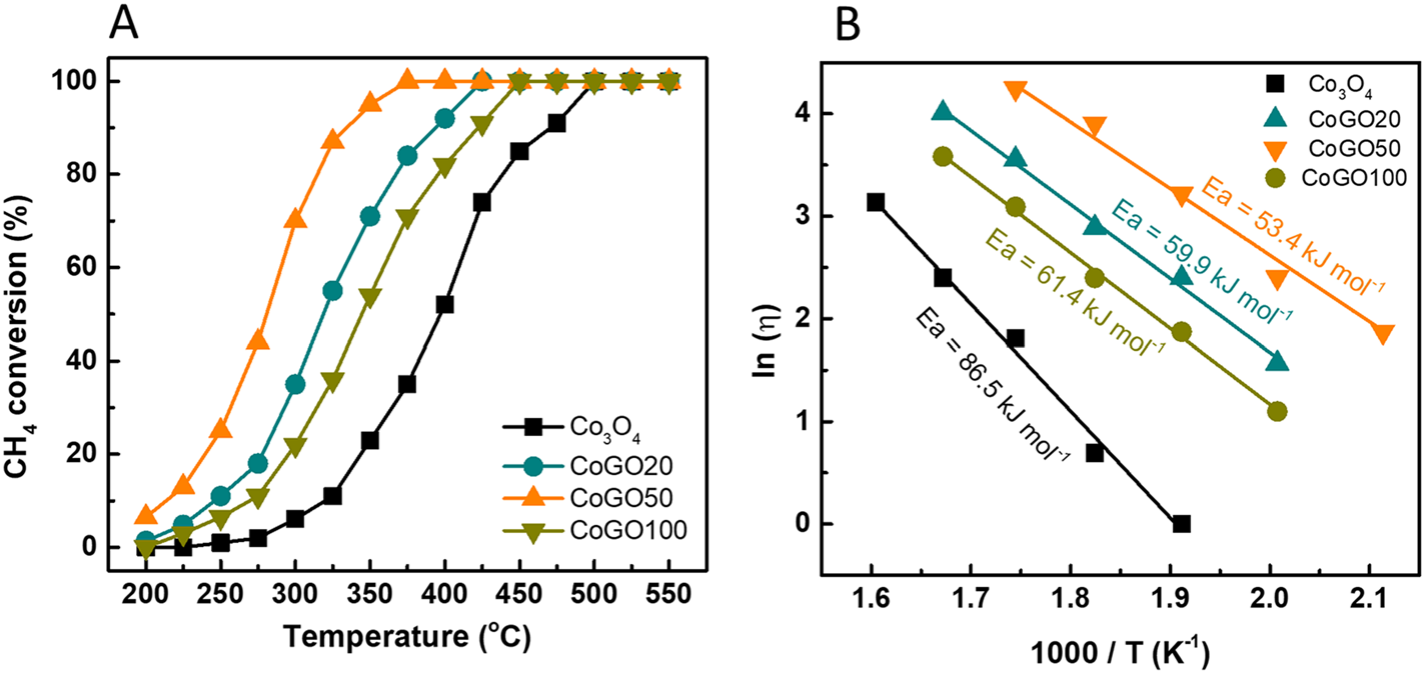
(**A**) Methane conversion over Co_3_O_4_, CoGO20, CoGO50 and CoGO100 catalysts and (**B**) Temperature dependence of CH_4_ conversion on various catalysts (1 vol% CH_4_, 10 vol% O_2_ and N_2_ balance, P = 1 bar, GHSV = 30,000 mL g^−1^ h^−1^).

Methane catalytic combustion on Co_3_O_4_ and CoGOx (x = 20, 50 and 100) catalysts were tested in a micro-fixed-bed reactor at 200 to 500 °C. The reaction was carried out with a feeding conditions of 1 vol% of CH_4_, 10 vol% of O_2_ and 89 vol% of N_2_ and GHSV = 30,000 mL g^−1^ h^−1^. With temperature increasing, as shown in Fig. 5A, the methane conversion on these catalysts increased from 0 to 100% in the range of 200 to 500 °C. However, CoGOx catalysts exhibit higher activities at low temperature than Co_3_O_4_. In detail, the temperature for 100% conversion of methane (T_100_) is 500 °C for Co_3_O_4_, which significantly decreases to 425, 400 and 450 °C for CoGO20, CoGO50 and CoGO100 catalysts, respectively. Similarly, the temperatures for methane conversion of 10% (T_10_), 50% (T_50_) and 90% (T_90_) of CoGOx are much lower than that of Co_3_O_4_ (Table 2). CoGO50 has the highest activity for methane conversion with the lowest T_100_, T_90_, T_50_ and T_10_ and T_100_, which are 100 °C, 105 °C, 103 °C and 105 °C lower than the corresponding values of Co_3_O_4_. It demonstrates that GO involved preparation is significantly improve the reactivity of CoGO50 through forming surface defects.

**Table 2:** Reaction temperature for obtaining 10%, 50%, 90% and 100% methane conversion (1 vol% CH_4_, 10 vol% O_2_ and N_2_ balance, P = 1 bar, GHSV = 30,000 mL g^−1^ h^−1^).

Catalysts	T_10_ (^o^C)	T_50_ (^o^C)	T_90_ (^o^C)
Co_3_O_4_	323	398	475
CoGO20	245	320	395
CoGO50	218	295	370
CoGO100	274	345	425

The apparent activation energy (*E*_a_) of CH_4_ oxidation over the catalysts was also calculated via the Arrhenius plots (Fig. 5B). The order trend of *E*_a_ for CH_4_ oxidation on these catalysts is followed as Co_3_O_4_ (86.5 kJ mol^−1^) > CoGO100 (61.4 kJ mol^−1^) > CoGO20 (59.9 kJ mol^−1^) > CoGO50 (53.4 kJ mol^−1^), which means CH_4_ was most easily activated and oxidized by CoGO50. It is well consistent with the reactivities of these catalysts. The reactivity of CoGO50 for methane combustion was compared with the reported results over various catalysts (Table S2). CoGO50 is more active than the cobalt oxide catalyst and even comparable to the noble metal-based catalysts under the similar conditions.

**Figure 6:**
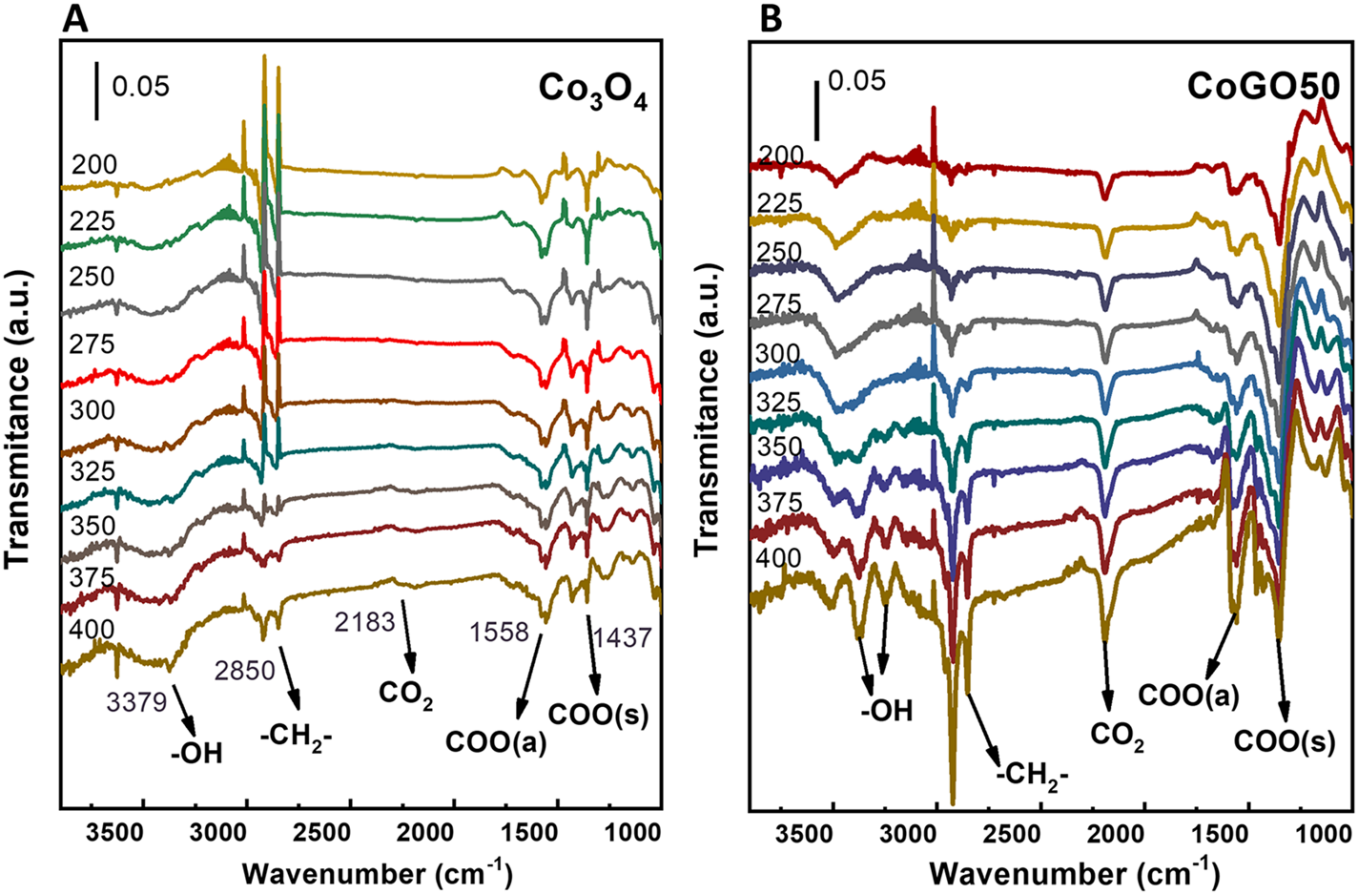
In situ DRIFTS spectra of (**A**) Co_3_O_4_ and (**B**) CoGO50 for methane combustion at different temperature (1 vol% CH_4_, 10 vol% O_2_ balanced with N_2_).

To investigate the temperature sensibility of methane conversion, both Co_3_O_4_ and CoGO50 were comparatively measured by using in-situ diffuse reflectance infrared Fourier transform spectra (DRIFTS) with simulated feeding at different temperature (Fig. 6). The band at 2880 cm^−1^ represents the C–H stretching, which is ascribed to the adsorbed and/or the activated CH_4_ on the catalyst^23,39^. The bands at 1560 cm^−1^ and 1420 cm^−1^ can be assigned to the asymmetric and symmetric stretching vibration of intermediate carbonates (ν_as_CO_3_^2+^ and ν_s_CO_3_^2+^), respectively^40^. The band at 3380 cm^−1^ could be assigned to the stretching vibration of hydroxyl, which may reflect the end product of adsorbed water^23,39^. The band at 2200 cm^−1^ is ascribed to the end product CO_2_^39^. For both samples, the intensities of all signals from CH_4_ adsorption/activation, intermediates and end products increase with the reaction temperature, in line with the real reaction results. However, the DRIFTS spectra of CoGO50 show stronger signals of intermediates and products at low temperature in compared with that of Co_3_O_4_ (Fig. 5A,B). The signal of CO_2_ can be observed from CoGO50 even at 200 °C, while the temperature with visible CO_2_ peak is 325 °C for Co_3_O_4_. This is well consistent with the real reaction tests that CoGO50 have the same reactivity for methane at the temperature more than 100 °C lower than Co_3_O_4_.

**Figure 7:**
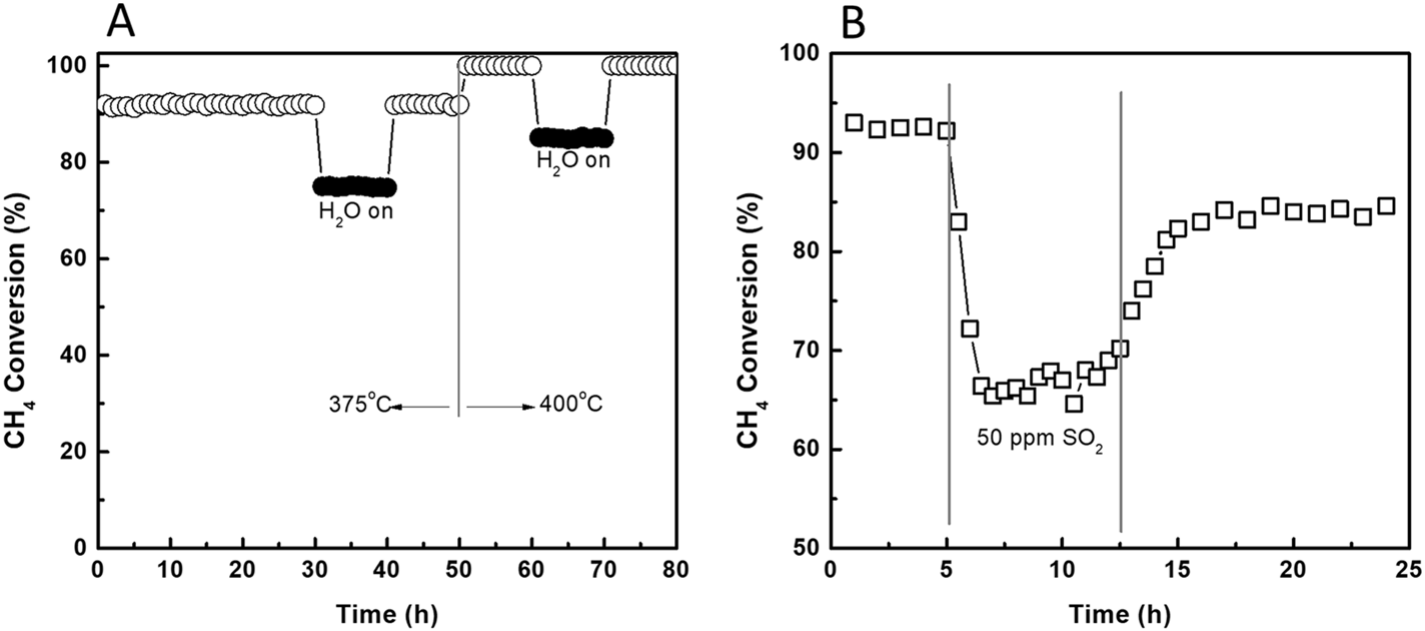
(**A**) The effects of water vapor (10 vol%) on the methane combustion performance of CoGO50 and (**B**) The effects of 50 ppm SO_2_ on the methane conversion over CoGO50 sample at 350 °C (1 vol% CH_4_, 10 vol% O_2_ and N_2_ and GHSV = 30,000 mL g^−1^ h^−1^).

The effects of impurities of feed gas on the performance of CoGO50 were tested by using water vapor and SO_2_, respectively. Figure 7A shows the stability of CoGO50 for methane combustion at 375 and 400 °C. At 375 °C, the CH_4_ conversion remain stable around 92%. With 10% water vapor feeding, the CH_4_ conversion decreased rapidly and kept stable on 75%. This indicates that vapor environment has a suppression effect on the CH_4_ oxidation. On the one hand, water vapor may adsorb and cover partial of active sites of catalyst to form Co(OH)_2_^41^. On the other hand, the oxidation of CH_4_ to CO_2_ and H_2_O may be thermodynamically limited as the feeding water is one of the products. The conversion can be 100% recovered after switching the vapor off. At 400 °C, the CH_4_ conversion presents the similar change tendency in the conditions of introduce/shut-down10% vapor, which first declined from 100 to ~ 85% and then returned to 100%.

The poisoning effects of sulphur dioxide on the catalyst was also tested over CoGO50, in which 50 ppm SO_2_ was introduced into the reaction system at 350 °C (Fig. 7B). Upon SO_2_, the methane conversion rapidly decreased from 91 to ~ 65% in 3 h. It was then slowly increased to ~ 70% in the following 5 h. After switching SO_2_ off and purging with N_2_, the CH_4_ conversion slightly increased to ~ 85% in 4 h and then kept stable, suggesting that more than 93% of original reactivity was recovered. This indicates that SO_2_ tends to impact the activity of CoGO50, irreversibly. SO_2_ can strongly adsorb on the catalyst via the reactions of Co_3_O_4_ + SO_2_ → Co_3_O_4_·SO_2_, which may further form sulphates through the reactions of 2Co_3_O_4_·SO_2_ + O_2_ → 2Co_3_O_4_·SO_3_ or Co_3_O_4_·SO_3_ → Co_3_(SO_4_)_4_^42^.

## Discussion

In summary, the 2D Co_3_O_4_ nanoplates containing surface defects have been demonstrated by using graphene oxide flakes not only as shaping templates but also as sacrifice agent for defect formation. The resultant CoGO50 exhibited a superior low-temperature reactivity for the methane combustion to CO_2_ and H_2_O in comparison with the GO-free Co_3_O_4_ catalyst, in which CoGO50 offered T_100_, T_90_ and T_50_ at least 100 °C lower than that of common Co_3_O_4_. Moreover, CoGO50 posted the completely recoverable reactivity upon the intermittent feeding of water vapor in the methane combustion. This work demonstrated that GO plays as 2D confine template to form smaller and thinner nanoplates. Importantly, the GO derivative (i.e. thermal reduced GO) plays as a surface reductant during the formation of Co_3_O_4_ nanoplates, which significantly affects the surface valence state, active oxygen species and surface oxygen vacancies of the resultant Co_3_O_4_ nanoplates. Consequently, this work provides a facile method to construct 2D catalyst with defective surface and enhanced oxidation reactivity.

## Methods

### Catalyst preparation

Graphene oxide (GO) powder was supplied by Xianfeng Nanomaterial Co. (China) and used without further treatments. Co(NO_3_)_2_ (> 99%) and KOH (> 99%) were supplied by Aladdin Co. Certain weight of GO (20, 50 or 100 mg) was dispersed in 100 mL deionized water with sonication for 0.5 h, followed by addition of 0.06 mol KOH. Co(NO_3_)_2_ aqueous solution (0.4 mol L^−1^, 50 mL) was then dropped into the GO alkaline solution by a constant flow pump under vigorous stirring to form dark grey precipitate. After the mixing of GO alkaline solution and Co(NO_3_)_2_ solution, the resulting solution was further aged for 1 h at room temperature. Then the precipitate was separated by centrifugation and washed with deionized water for three times. Finally, the precipitate was dried at 110 °C in vacuum and calcinated at 360 °C in air for 6 h, in sequence. Based on the amount of GO used in the preparation, the resultant catalysts were named as CoGO20, CoGO50 and CoGO100, respectively. As a control, the catalyst Co_3_O_4_ was synthesized under the same conditions without the addition of GO.

### Methane catalytic combustion

The catalytic combustion of methane was tested in a fixed bed quartz tube reactor (ID = 6 mm, L = 500 mm) at atmospheric pressure^43^. 100 mg catalyst (40–60 mesh) was packed in the middle of reactor with quartz wool layers on both ends of reactor. Mixture gas containing 1 vol% CH_4_, 10 vol% O_2_ and 89 vol% N_2_ was supplied by Pujiang Gas Co. The above feed gas was introduced to reactor with a flow rate of 50 mL min^−1^, which corresponds to a gas hourly space velocity (GHSV) of 30,000 mL g^−1^ h^−1^. The temperature was increased stepwise from 200 to 550 °C at a ramp of 5 °C min^−1^. The temperature dependence of CH_4_ conversion was tested with an interval of 25 °C in the range of 200–550 °C. The reaction products were analysed by a micro gas chromatograph (INFICON 3000) equipped with MS5A and Plot Q columns and TCD detector. Before analysis, the reaction temperature at each step was stabilized at least 30 min. To investigate the effects of impurities on the catalytic performance, 10 vol% of water vapor was introduced into the reactor together with feed gas mixture. For the tests of sulphur tolerance, a mixed gas of 50 ppm SO_2_, 1 vol% CH_4_, 10 vol% O_2_ balanced with high purity N_2_ was used with a flow rate of 50 mL min^−1^.

### Characterization

The morphology of the catalyst samples was measured by a transmission electron microscope (TEM, JEM-2100, 200 kV) and a scanning electron microscope (SEM, Zeiss SUPRA 55 SAPPHIRE, 2–20 kV). The structure of Co_3_O_4_ based catalysts was recorded by X-ray diffraction (XRD, Rigaku Ultima IV) with Cu Kα radiation (λ = 0.15406 nm, 40 kV, 40 mA). The specific surface area and pore size distribution of catalysts were measured by N_2_-sorptions on an automatic micropore physisorption analyser (TriStar II 3020). Specific surface areas of the products were calculated by the Brunauer–Emmett–Teller (BET) method and pore sizes were calculated using the Barrett-Joyner-Halenda (BJH) method. Raman spectroscopy measurements were performed using a Renishaw Raman spectrometer using a 12.5 mW laser source at an excitation wavelength of 532 nm. The near-surface chemical information of catalysts was analysed by X-ray photoelectron spectroscopy (XPS, Thermo Scientific K-Alpha) using Al Kα (hν = 1486.6 eV) as the excitation source. XPS peak positions were corrected with the help of the C 1s peak at 284.8 eV. The thermal behaviours of catalysts and the catalyst precursor were analysed by a thermogravimetric analyser with a mass spectrometer (TGA-MS, NETZSCH, STA 449F3) operated from 60 to 1000 °C with heating rate of 5 °C min^−1^ under an air flow (20 mL min^−1^). Hydrogen temperature-programmed reduction (H_2_-TPR) was carried out on a chemical adsorption analyser (Micromeritics AutoChem II 2920) equipped with a thermal conductivity detector (TCD). In situ diffuse reflectance infrared Fourier transform spectra (DRIFTS) of Co_3_O_4_ and CoGO 50 catalysts were recorded by a Bruker Vertex 70 spectrometer in the simulated reactant (1% CH_4_ + 21% O_2_ + N_2_ balance, 30 mL min^−1^) at the temperature range of 200 to 400 °C with the interval of 25 °C.

## Supplementary Information

Supplementary Information.
