# Supplementary material for: Low-temperature combustion of methane over graphene templated Co3O4 defective-nanoplates
Source: Sci Rep. 2021 Jun 15;11:12604. doi: 10.1038/s41598-021-92165-4 (PMC8206361; doi:10.1038/s41598-021-92165-4)
Supplement: Supplementary file 1 — Supplementary Information. [file 41598_2021_92165_MOESM1_ESM.docx]

Supporting Information

Low-temperature combustion of methane over graphene templated Co_3_O_4_ defective-nanoplates

Dian Gong and Gaofeng Zeng*


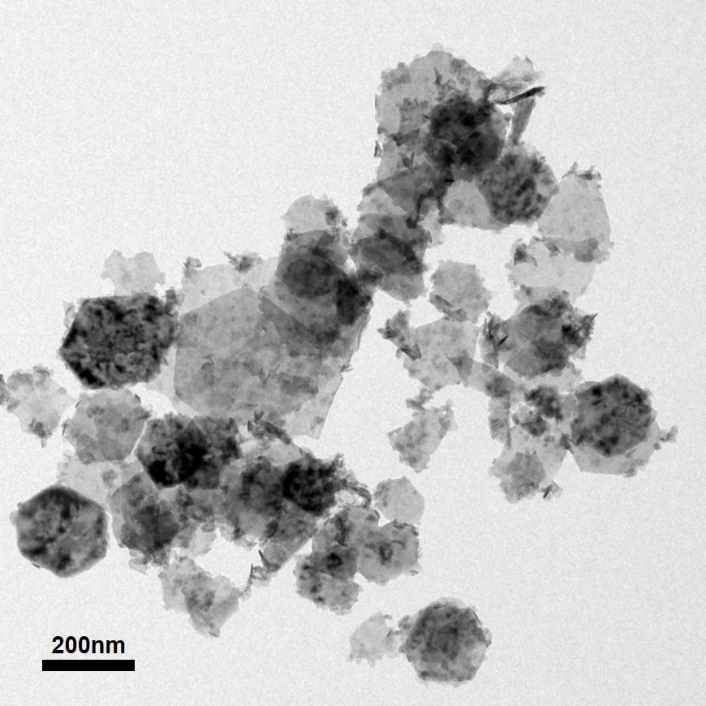


Figure S1. TEM image of CoOOH


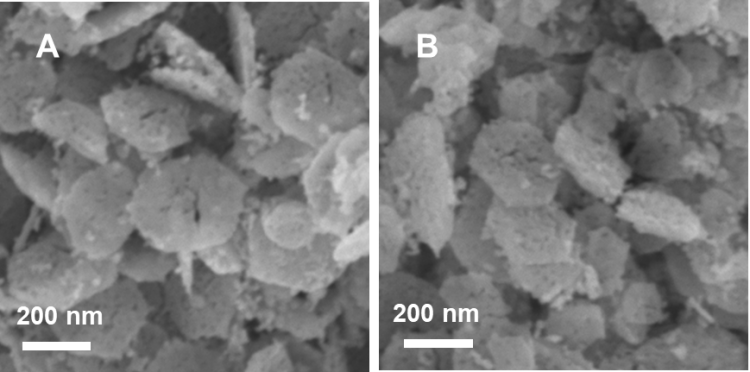


Figure S2. SEM images of (A) Co_3_O_4_ without GO addition and (B) CoGO20


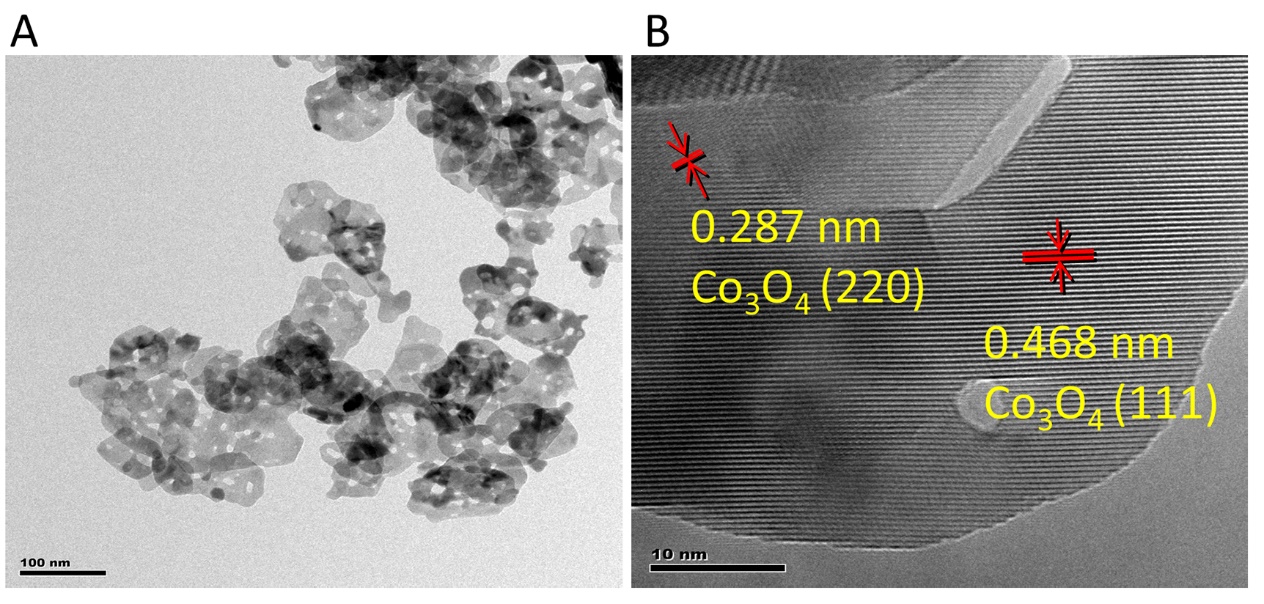


Figure S3. TEM (A) and HRTEM (B) images of Co_3_O_4_.

Figure S4. TG-DSC curves of CoGO50.


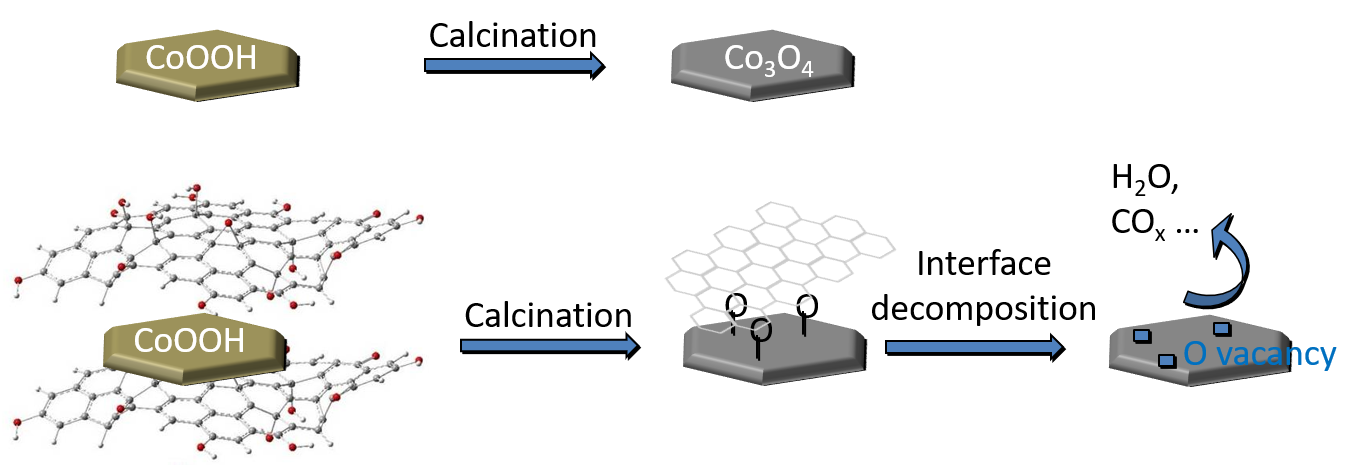


Figure S5. Illustration of the interface reaction in the CoGO catalysts (It was drawn by using Microsoft Office 365 with the version of 13901.20336 and URL of https://www.microsoft.com/zh-cn/microsoft-365).


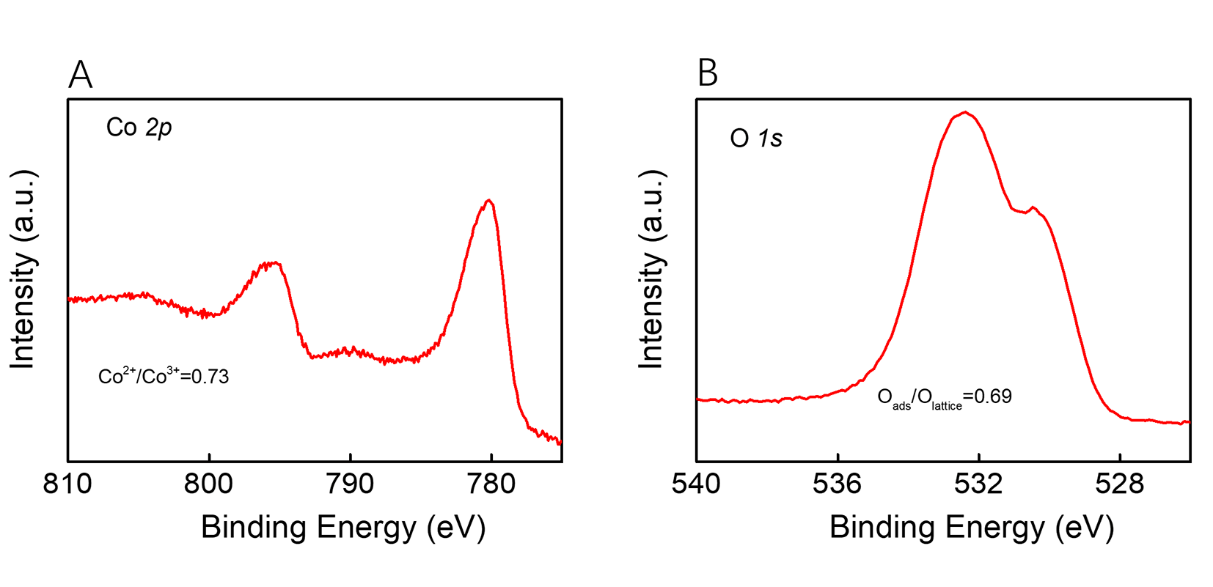


Figure S6. XPS Co 2p (A) and O 1s (B) of the spent CoGO50.

Table S1. BET surface areas, pore volumes and pore diameter of the samples.

| Sample | BET surface area  (m^2^g^-1^) | Pore volume  (cm^3^g^-1^) | Pore diameter  (nm) |
| --- | --- | --- | --- |
| Co_3_O_4_ | 31.5 | 0.23 | 29.1 |
| CoGO50 | 39.7 | 0.31 | 39.7 |
| CoGO100 | 43.2 | 0.28 | 30.5 |

Table S2 The catalytic performance comparison of CoGO50 for the methane combustion with the literature results.

| Catalysts | Metal  (wt %) | Feed | Space velocity  (mL·g^-1^·h^-1^) | Specific rates  at 250°C  (μmol·g_cat_^-1^·s^-1^) | T_50_  (°C) | T_90_  (°C) | Note |
| --- | --- | --- | --- | --- | --- | --- | --- |
| CoGO50 | 0 | 1% CH_4_, 10% O_2_, in N_2_ | 30,000 | 0.930 | 295 | 370 | This work |
| Co_3_O_4_(30 wt.%)–CeO_2_ | 0 | 0.3 vol.% of CH_4_ + 0.6 vol.%  O_2_ in He | 60,000 | 0.891 | 471 | 520 | ^1^ |
| Pd/Co_3_O_4_ (LT) | 3.21 | 1% CH_4_, 10% O_2_, in N_2_ | 30,000 | 0.949 | 277 | 314 | ^2^ |
| Pd/Co_3_O_4_ | 2.00 | 2% CH_4_ in air | 24,000 | 1.127 | 259 | 272 | ^3^ |
| Pd/Co_3_O_4_ | 5.00 | 2% CH_4_ in air | 24,000 | 5.194 | 236 | 248 | ^3^ |
| Au/ Co_3_O_4_ | 10.00 | 0.3% CH_4_, 2.4% O_2_ in He | 60,000 | 0.337 | 364 | ~475 | ^4^ |
| Pd/SnO_2_ on ceramic monolith | 8.70 | 1% CH_4_ in air | 41,000 h^-1^ | ~5.443 | ~268 | ~320 | ^5^ |
| Pd/Sn_0.4_Zr_0.6_O_2_ | 2.00 | 1% CH_4_ in air | 33,000 | ~0.164 | 329 | 378 | ^6^ |
| Pd/LaMnO_3_ | 1.75 | 1% CH_4_ in air | 8,333 | ~0.949 | ~427 | ~491 | ^7^ |
| Pd/HMS | 1.00 | 0.3% CH_4_, 2.4% O_2_ in He | 60,000 | ~0.000 | 341 | ~400 | ^8^ |

Reference

1. Liotta LF, Di Carlo G, Pantaleo G, Venezia AM, Deganello G. Co3O4/CeO2 composite oxides for methane emissions abatement: Relationship between Co3O4-CeO2 interaction and catalytic activity. *Applied Catalysis B-Environmental* **66**, 217-227 (2006).

2. Yang N, Ni S, Sun Y, Zhu Y. A facial strategy to synthesize Pd/Co3O4 nanosheets with enhanced performance for methane catalytic oxidation. *Molecular Catalysis* **452**, 28-35 (2018).

3. Hu LH, Peng Q, Li YD. Low-temperature CH4 Catalytic Combustion over Pd Catalyst Supported on Co3O4 Nanocrystals with Well-Defined Crystal Planes. *Chemcatchem* **3**, 868-874 (2011).

4. Liotta LF, Di Carlo G, Longo A, Pantaleo G, Venezia AM. Support effect on the catalytic performance of Au/Co3O4-CeO2 catalysts for CO and CH4 oxidation. *Catalysis Today* **139**, 174-179 (2008).

5. Kikuchi R, Maeda S, Sasaki K, Wennerstrom S, Ozawa Y, Eguchi K. Catalytic activity of oxide-supported Pd catalysts on a honeycomb for low-temperature methane oxidation. *Applied Catalysis a-General* **239**, 169-179 (2003).

6. Lin W, Lin L, Zhu YX, Xie YC, Scheurell K, Kemnitz E. Novel Pd/SnxZr1-xO2 catalysts for methane total oxidation at low temperature and their O-18-isotope exchange behavior. *Applied Catalysis B-Environmental* **57**, 175-181 (2005).

7. Giebeler L, Kiessling D, Wendt G. LaMnO3 perovskite supported noble metal catalysts for the total oxidation of methane. *Chemical Engineering & Technology* **30**, 889-894 (2007).

8. Venezia AM, Murania R, Pantaleo G, Deganello G. Pd and PdAu on mesoporous silica for methane oxidation: Effect of SO2. *Journal of Catalysis* **251**, 94-102 (2007).
